# Clinicopathological Spectrum of Adult Glomerulonephritis With Predominant C3 Deposition: A Four-Case Series Favoring Infection-Related Glomerulonephritis

**DOI:** 10.7759/cureus.117432

**Published:** 2026-10-04

**Authors:** Mohammed Hamzaoui, Imane Machmachi, Taoufik Harmouch, Intissar Haddiya, Yassamine Bentata

**Affiliations:** 1 Nephrology, Dialysis and Kidney Transplantation Unit, Mohammed VI University Hospital, Mohammed First University, Faculty of Medicine, Oujda, MAR; 2 Laboratory of Epidemiology, Clinical Research and Public Health, Faculty of Medicine, Mohammed First University, Oujda, MAR; 3 Anatomopathology, Harmouch Pathology Laboratory, Fez, MAR

**Keywords:** c3 deposition, c3 glomerulopathy, infection-related glomerulonephritis, post-infectious glomerulonephritis, renal biopsy

## Abstract

Infection-related glomerulonephritis (IRGN) in adults is a heterogeneous clinicopathological entity with generally less favorable renal outcomes than classic post-streptococcal glomerulonephritis in children. Predominant C3 deposition on kidney biopsy can pose a diagnostic challenge because it may closely mimic C3 glomerulopathy (C3G), particularly when the infectious trigger is remote, partially treated, or not well documented. We report a series of four adults with biopsy-proven glomerulonephritis showing predominant C3 deposition and clinicopathological features favoring an infection-related process. Clinical presentations were heterogeneous, ranging from acute kidney injury with nephrotic syndrome and crescentic glomerulonephritis to persistent microscopic hematuria and subnephrotic proteinuria with preserved kidney function. Kidney biopsy demonstrated diverse histopathological patterns, while immunofluorescence consistently showed predominant C3 deposition with absent or trace immunoglobulin staining. Renal function improved substantially in two patients and remained preserved in two patients, although long-term follow-up was incomplete in one patient. Cases 2 and 4 represented the main diagnostic challenge because of persistent urinary abnormalities in the absence of a documented active infection and incomplete complementary investigations, and C3G could not be definitively excluded. This case series highlights the heterogeneous clinicopathological spectrum of adult glomerulonephritis with predominant C3 deposition and emphasizes the importance of integrating clinical history, microbiological investigations, complement evaluation, kidney biopsy findings, and clinical evolution to distinguish IRGN from C3G. Accurate clinicopathological correlation is essential because this distinction has important therapeutic and prognostic implications.

## Introduction

Infection-related glomerulonephritis (IRGN) is a glomerular disease that develops during or following an infectious process. Although historically described as post-streptococcal glomerulonephritis in children, the epidemiology of IRGN has changed considerably over recent decades. Adult cases are increasingly recognized, particularly among individuals with diabetes mellitus, advanced age, chronic comorbidities, or immunocompromised states. In adults, *Staphylococcus aureus* is among the most frequently implicated pathogens, and renal outcomes are generally less favorable than those of classic post-streptococcal glomerulonephritis in children [1-3].

The diagnosis of IRGN relies on the integration of clinical history, microbiological evidence, serum complement evaluation, and kidney biopsy findings. The classic histopathological pattern consists of diffuse endocapillary proliferative glomerulonephritis with prominent C3 deposition on immunofluorescence and subepithelial hump-shaped electron-dense deposits on electron microscopy, when available [1,2]. However, the pathological spectrum is broader than previously recognized and may include mesangial proliferative, exudative, and crescentic forms. Moreover, prior antibiotic exposure, delayed presentation, or the absence of an active infection at the time of kidney biopsy may complicate the diagnosis [1,2].

A major diagnostic challenge arises when kidney biopsy demonstrates predominant C3 deposition with absent or only trace immunoglobulin staining. In such cases, distinguishing IRGN from C3 glomerulopathy (C3G) may be particularly difficult because both entities share overlapping clinical, histological, and immunopathological features [4-6]. Nevertheless, distinguishing these disorders is important because their underlying mechanisms, therapeutic approaches, and long-term renal outcomes may differ. Complement studies, microbiological investigations, and electron microscopy may assist in establishing the diagnosis; however, these investigations are not always available in routine clinical practice [4-8].

Reports specifically addressing adult infection-related glomerulonephritis with predominant C3 deposition remain limited. Herein, we present a four-case series of adults with biopsy-proven glomerulonephritis showing predominant C3 deposition and clinicopathological features favoring an infection-related process. The cases illustrate a broad clinical and pathological spectrum, ranging from acute kidney injury with nephrotic syndrome and crescentic glomerulonephritis to persistent microscopic hematuria and subnephrotic proteinuria with preserved kidney function. We also highlight the diagnostic challenges in distinguishing infection-related glomerulonephritis from C3 glomerulopathy, particularly when the infectious trigger is remote, partially treated, or not definitively documented.

## Case presentation

Case 1

A 36-year-old man with recently diagnosed type 1 diabetes mellitus, treated with a basal-bolus insulin regimen, was admitted to our tertiary nephrology department for evaluation of acute kidney injury (AKI) associated with nephrotic syndrome. One month before admission, he had been hospitalized for a pulmonary infection and received antibiotic therapy, with reported clinical and biological improvement. During this hospitalization, a catheter-associated urinary tract infection caused by *Staphylococcus aureus *was documented; the patient had been catheterized at admission. He initially received intravenous ceftriaxone, which was discontinued because of a shortage of the drug at our institution. Because of persistent systemic inflammation and a suspected pulmonary infectious focus, antibiotic therapy was switched to intravenous ciprofloxacin and subsequently to intravenous levofloxacin combined with oral amoxicillin-clavulanate. The inflammatory response gradually improved, with a decline in C-reactive protein levels. Despite antimicrobial therapy, kidney function remained impaired, prompting referral to our tertiary nephrology center.

At presentation, blood pressure was 130/69 mmHg. Physical examination revealed bilateral lower-limb edema extending to the thighs and bilateral basal crackles, while urine output remained preserved at approximately 2 L/day.

Laboratory investigations demonstrated severe AKI, with a serum creatinine of 289 µmol/L and serum urea of 38.3 mmol/L. Serum albumin was markedly decreased at 17 g/L, with a total serum protein level of 52 g/L. The urine protein-to-creatinine ratio was 2.99 g/g and was associated with microscopic hematuria. The combination of marked hypoalbuminemia, generalized edema, and heavy proteinuria was clinically consistent with nephrotic syndrome. Inflammatory markers were markedly elevated, with a C-reactive protein level of 123 mg/L and leukocytosis of 22.03 × 10⁹/L with neutrophil predominance. Urine culture grew *Staphylococcus aureus*, and serum complement levels were decreased (C3, 0.11 g/L; C4, 0.06 g/L).

Chest radiography subsequently demonstrated a right upper-lobe infectious focus with blunting of the costophrenic angle. Chest computed tomography later revealed bilateral nodular parenchymal consolidations, some with necrotic features and air bronchograms, bilateral pleural effusions, and focal ground-glass opacities (Figure 1). These findings were considered highly suggestive of an infectious pulmonary process. Ultrasound-guided thoracentesis demonstrated an exudative pleural effusion. Transthoracic echocardiography performed to exclude infective endocarditis showed no valvular vegetations.

**Figure 1:**
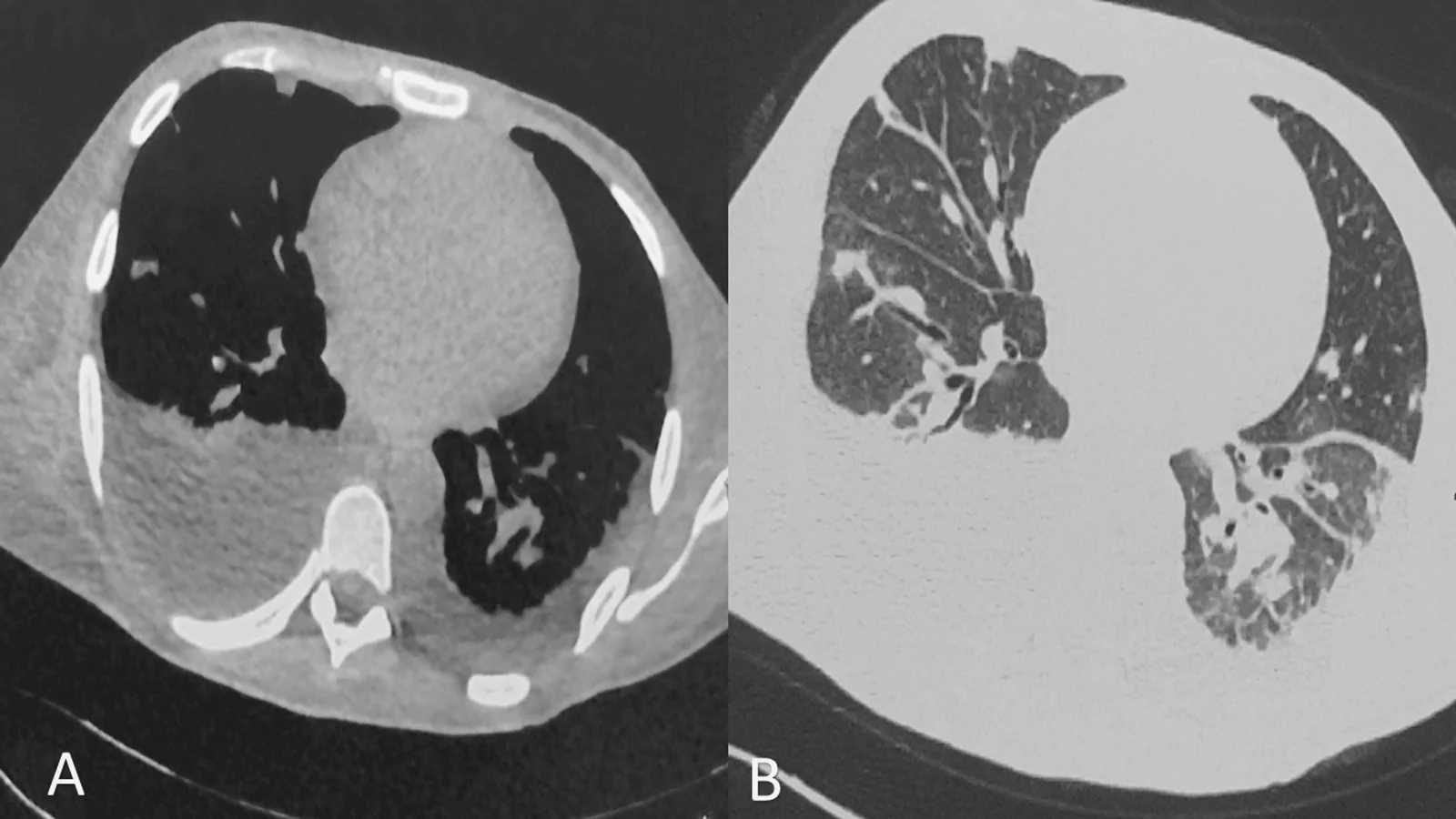
Chest computed tomography findings in Case 1 (A) Representative axial chest computed tomography image in the mediastinal window showing bilateral pleural effusions. (B) Representative axial chest computed tomography image in the lung window showing bilateral pulmonary air-space consolidations, with associated air bronchograms and areas of parenchymal involvement.

An ultrasound-guided kidney biopsy was subsequently performed. Light microscopy demonstrated diffuse global endocapillary proliferative glomerulonephritis with extracapillary proliferation involving two glomeruli. Mild inflammatory tubulointerstitial injury affecting less than 10% of the cortical area was present. No double-contour formation or Congo red-positive deposits were identified on light microscopy.

Immunofluorescence demonstrated diffuse granular mesangial C3 deposition with only trace mesangial IgG staining and no IgA, IgM, C1q, kappa, or lambda deposits. These clinicopathological findings were compatible with IRGN, consistent with the histopathological interpretation. Representative light microscopy findings are shown in Figure 2.

**Figure 2:**
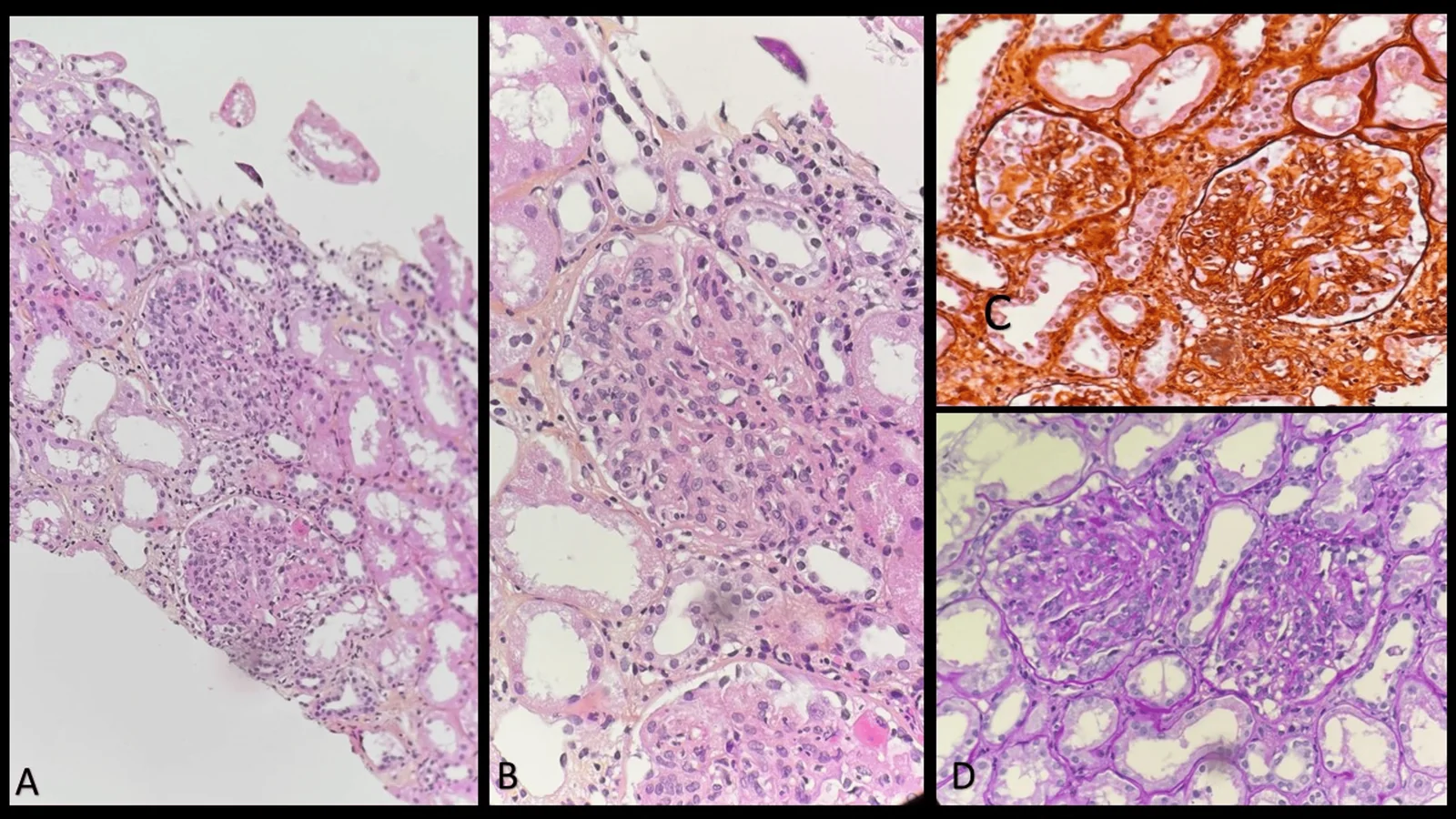
Representative light microscopy findings in Case 1 (A) Low-power hematoxylin and eosin (H&E) staining (×100) showing several glomeruli with diffuse glomerular hypercellularity and mild tubulointerstitial inflammatory involvement. (B) High-power H&E staining (×400) demonstrating increased glomerular cellularity consistent with endocapillary proliferative glomerulonephritis. (C) Reticulin staining (×200) highlighting the glomerular and capillary architecture. (D) Periodic acid–Schiff (PAS) staining (×200) highlighting the glomerular architecture and capillary loops.

The patient received supportive management, including diuretics and prophylactic anticoagulation in the setting of severe hypoalbuminemia. Neither corticosteroids nor other immunosuppressive therapy was administered. Kidney function improved progressively during follow-up, with serum creatinine decreasing to 133 µmol/L after one month and 88 µmol/L after three months. Proteinuria and microscopic hematuria completely resolved, serum albumin increased to 31 g/L, and serum C3 normalized. The patient achieved complete clinical and laboratory remission, with recovery of kidney function, resolution of proteinuria and microscopic hematuria, and normalization of serum complement levels.

Case 2

A 31-year-old woman (gravida 2, para 2) with a history of recurrent pharyngitis was referred to our tertiary nephrology department for evaluation of persistent microscopic hematuria and proteinuria. Urinary abnormalities were first identified during the first trimester of pregnancy, several months before presentation, when she presented with a hypertensive emergency without clinical or biological evidence of preeclampsia. Initial investigations showed a urine protein-to-creatinine ratio of 0.6 g/g, which subsequently persisted at 0.8 g/g and was associated with microscopic hematuria. She had no history of nephrolithiasis, nephrotoxic drug exposure, or familial kidney disease.

At presentation, blood pressure was 149/91 mmHg. Physical examination was unremarkable, with preserved urine output, no peripheral edema, and no clinical signs of fluid overload. Kidney function was preserved, with a serum creatinine of 49 µmol/L. Laboratory investigations demonstrated persistent proteinuria (urine protein-to-creatinine ratio, 0.8 g/g) associated with microscopic hematuria. C-reactive protein was mildly elevated at 11 mg/L, and urine culture was sterile. Serum complement levels (C3 and C4) were within the normal range. Because the urinary abnormalities persisted despite preserved kidney function, an ultrasound-guided kidney biopsy was performed.

Light microscopy demonstrated diffuse global mesangial proliferative glomerulonephritis with exudative features and mild inflammatory interstitial fibrosis involving approximately 5% of the cortical area. No endocapillary or extracapillary proliferation or double-contour formation was identified. No extramembranous or other abnormal deposits were observed. Congo red staining was negative. Numerous red blood cell casts were observed within the renal tubules. The renal arteries and arterioles appeared normal.

Immunofluorescence performed on a fresh biopsy core containing six glomeruli demonstrated diffuse, global granular mesangial C3 deposition of moderate-to-marked intensity, associated with only trace mesangial IgM staining and no IgG, IgA, C1q, kappa, or lambda deposits. Moderate segmental and focal fibrinogen deposition was also observed. The pathology report interpreted the findings as chronic non-proliferative glomerulonephritis with C3 deposition and considered the findings compatible with either late post-infectious glomerulonephritis or C3 glomerulopathy. Representative light microscopy findings are shown in Figure 3.

**Figure 3:**
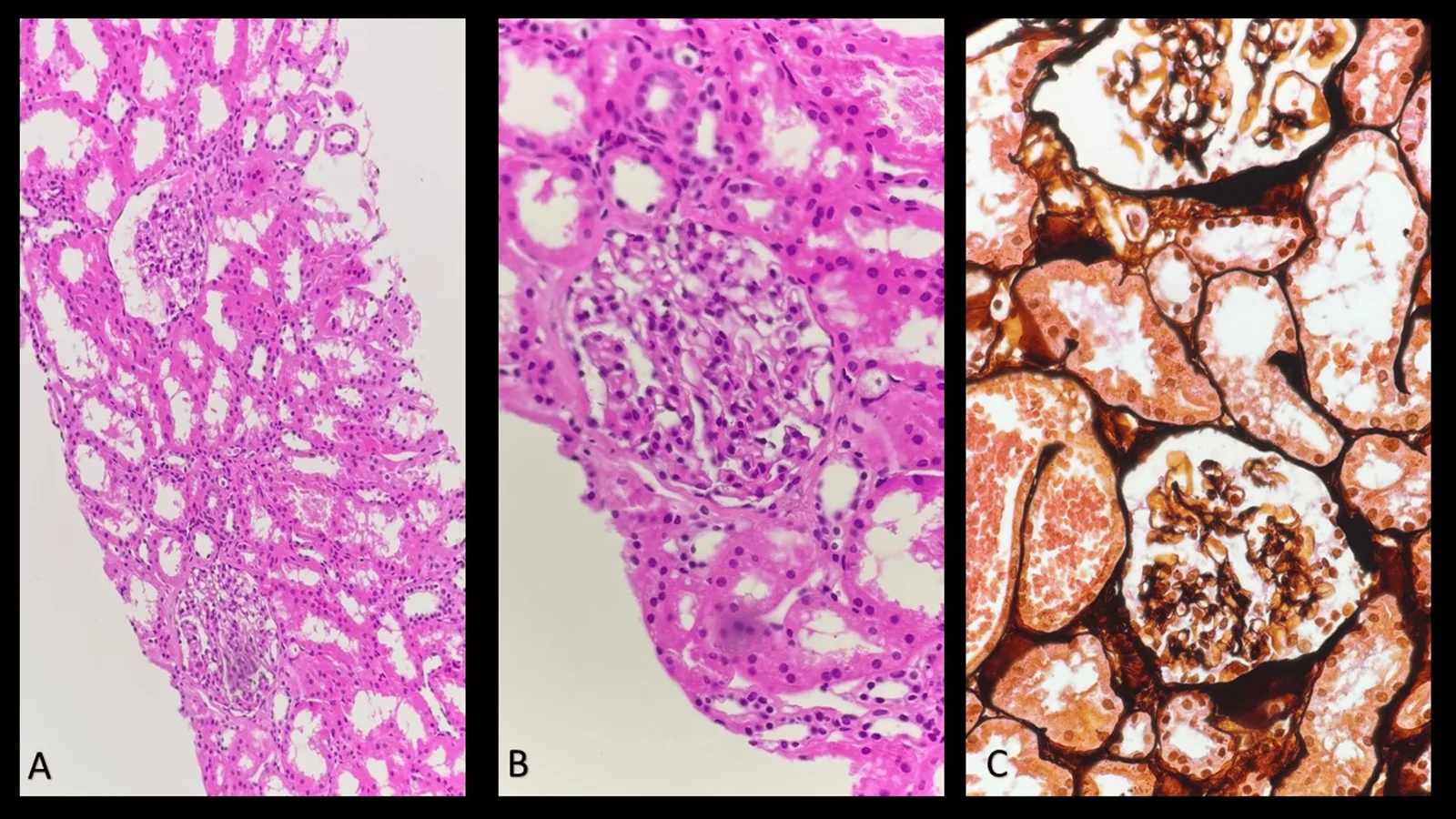
Representative light microscopy findings in Case 2 (A) Low-power hematoxylin and eosin (H&E) staining (×100) showing renal cortical tissue with glomeruli displaying diffuse hypercellularity and associated mild tubulointerstitial inflammatory changes. (B) High-power H&E staining (×400) demonstrating diffuse mesangial hypercellularity with exudative features. (C) Reticulin staining (×200) highlighting the glomerular architecture and capillary loops.

Given the history of recurrent pharyngitis, a remote or resolving infection-related process was considered clinically plausible; however, C3 glomerulopathy could not be definitively excluded.

The patient was treated with ramipril without corticosteroids, immunosuppressive therapy, or additional antibiotic therapy. At follow-up, blood pressure was 130/81 mmHg and kidney function remained preserved. However, urinary abnormalities persisted, with urine dipstick testing at subsequent follow-up showing 3+ blood and a urine protein-to-creatinine ratio of approximately 1.0 g/g.

Additional investigations, including CH50, anti-streptolysin O antibodies, anti-DNase B antibodies, and repeat serum complement measurements, were planned to further distinguish a remote or resolving infection-related process from C3 glomerulopathy. However, these investigations could not be completed because of financial and resource limitations. Consequently, although the overall clinicopathological findings favored a remote or resolving IRGN, C3 glomerulopathy could not be definitively excluded.

Case 3

A 43-year-old man with no previous history of diabetes mellitus, hypertension, cardiovascular disease, or kidney disease was referred to our tertiary nephrology department for evaluation of AKI. Two weeks before admission, he had been hospitalized for acute pansinusitis confirmed by computed tomography, which demonstrated complete opacification of the paranasal sinuses. He received an eight-day course of intravenous imipenem/cilastatin. Although his infectious symptoms improved following antibiotic therapy, inflammatory markers remained elevated and kidney function progressively deteriorated, with serum creatinine increasing from 194 µmol/L to 247 µmol/L, 318 µmol/L, and finally 361 µmol/L at presentation. During the initial evaluation, the patient also underwent contrast-enhanced thoracoabdominal and brain computed tomography.

At presentation, blood pressure was 120/65 mmHg. Physical examination revealed preserved urine output without peripheral edema, oliguria, or macroscopic hematuria. Laboratory investigations demonstrated severe AKI with a serum creatinine of 361 µmol/L, serum urea of 27.6 mmol/L, hyperkalemia (6.1 mmol/L), hypoalbuminemia (24 g/L), anemia (hemoglobin 89 g/L), leukocytosis (24.97 × 10⁹/L), thrombocytosis (783 × 10⁹/L), and an elevated C-reactive protein level (78 mg/L). The urine protein-to-creatinine ratio was 2.2 g/g, associated with persistent microscopic hematuria. Serum complement levels were within the normal range (C3, 1.27 g/L; C4, 0.21 g/L). Autoimmune investigations, including antinuclear antibodies, anti-neutrophil cytoplasmic antibodies (ANCA), anti-glomerular basement membrane antibodies, and anti-phospholipase A2 receptor antibodies (anti-PLA2R), were negative. The anti-streptolysin O antibody titer was <50 IU/mL, and serological tests for hepatitis B virus, hepatitis C virus, and human immunodeficiency virus were negative.

Computed tomography demonstrated kidneys of normal size and bilateral pleural effusions without deep lymphadenopathy. Given the rapidly progressive deterioration in kidney function, the principal differential diagnoses included rapidly progressive glomerulonephritis, ANCA-associated vasculitis, and IRGN. An ultrasound-guided kidney biopsy was therefore performed.

Light microscopy demonstrated crescentic glomerulonephritis, with circumferential extracapillary proliferation involving two of eight glomeruli examined. One glomerulus showed glomerular necrosis, while diffuse mesangial sclerosis was observed in the remaining glomeruli. Severe tubulointerstitial inflammatory injury was characterized by marked fibroedema with a dense and diffuse inflammatory infiltrate composed predominantly of mononuclear cells and numerous eosinophils. No vascular lesions were identified.

Immunofluorescence performed on two glomeruli demonstrated granular C3 deposition in the mesangium and along the glomerular capillary walls, whereas IgG, IgA, IgM, and C1q showed no significant deposits. Interstitial fibrin deposits were also observed. The biopsy was interpreted as extracapillary glomerulonephritis with granular C3 deposition, with a probable post-infectious origin and severe subacute interstitial lesions. No evidence of vasculitis was identified.

The patient initially received conservative management consisting of correction of electrolyte disturbances and supportive care for AKI. In view of the clinicopathological findings supporting an infection-related etiology and the subsequent improvement in kidney function with supportive care, neither corticosteroids nor immunosuppressive therapy was initiated, and renal replacement therapy was not required. An angiotensin-converting enzyme inhibitor was introduced after kidney biopsy once renal function had improved. Kidney function improved progressively during follow-up, with serum creatinine decreasing to 230 µmol/L at hospital discharge, 194 µmol/L after one month, 106 µmol/L after six months, and 88 µmol/L at the last available follow-up. Proteinuria regressed to trace levels, whereas microscopic hematuria persisted. The patient achieved near-complete renal recovery without dialysis or immunosuppressive therapy.

Case 4

A 46-year-old woman with no previous history of diabetes mellitus, hypertension, cardiovascular disease, or kidney disease was referred to our tertiary nephrology department for evaluation of persistent microscopic hematuria and proteinuria. Four months before admission, she developed erythematous skin lesions involving both lower limbs and was hospitalized in the Department of Internal Medicine for evaluation of a chronic inflammatory syndrome. Initial laboratory investigations revealed a urine protein-to-creatinine ratio of 1.68 g/g associated with microscopic hematuria, prompting referral for nephrological evaluation.

At presentation, blood pressure was 124/65 mmHg. Physical examination was unremarkable, with preserved urine output, no peripheral edema, and no signs of fluid overload. Kidney function was preserved, with a serum creatinine of 62 µmol/L. Laboratory investigations demonstrated persistent proteinuria (urine protein-to-creatinine ratio, 1.2 g/g) associated with microscopic hematuria, markedly elevated C-reactive protein (204 mg/L), mild anemia (hemoglobin 90 g/L), thrombocytosis (416 × 10⁹/L), and a sterile urine culture. Because urinary abnormalities persisted despite preserved kidney function, an ultrasound-guided kidney biopsy was performed.

Light microscopy demonstrated diffuse global mesangial proliferative glomerulonephritis with mesangial matrix expansion, without endocapillary or extracapillary proliferation. Mild tubulointerstitial inflammatory fibrosis involving less than 5% of the cortical area was present, whereas the renal vessels appeared unremarkable.

Immunofluorescence performed on a fresh biopsy core containing five glomeruli demonstrated diffuse and global granular mesangial C3 deposition of low-to-moderate intensity, associated with only trace mesangial IgM staining and no IgG, IgA, C1q, kappa, or lambda deposits. Fibrinogen deposition was absent. The pathology report considered the findings compatible with early or late post-infectious glomerulonephritis, although C3 glomerulopathy could not be formally excluded. Representative light microscopy findings are shown in Figure 4.

**Figure 4:**
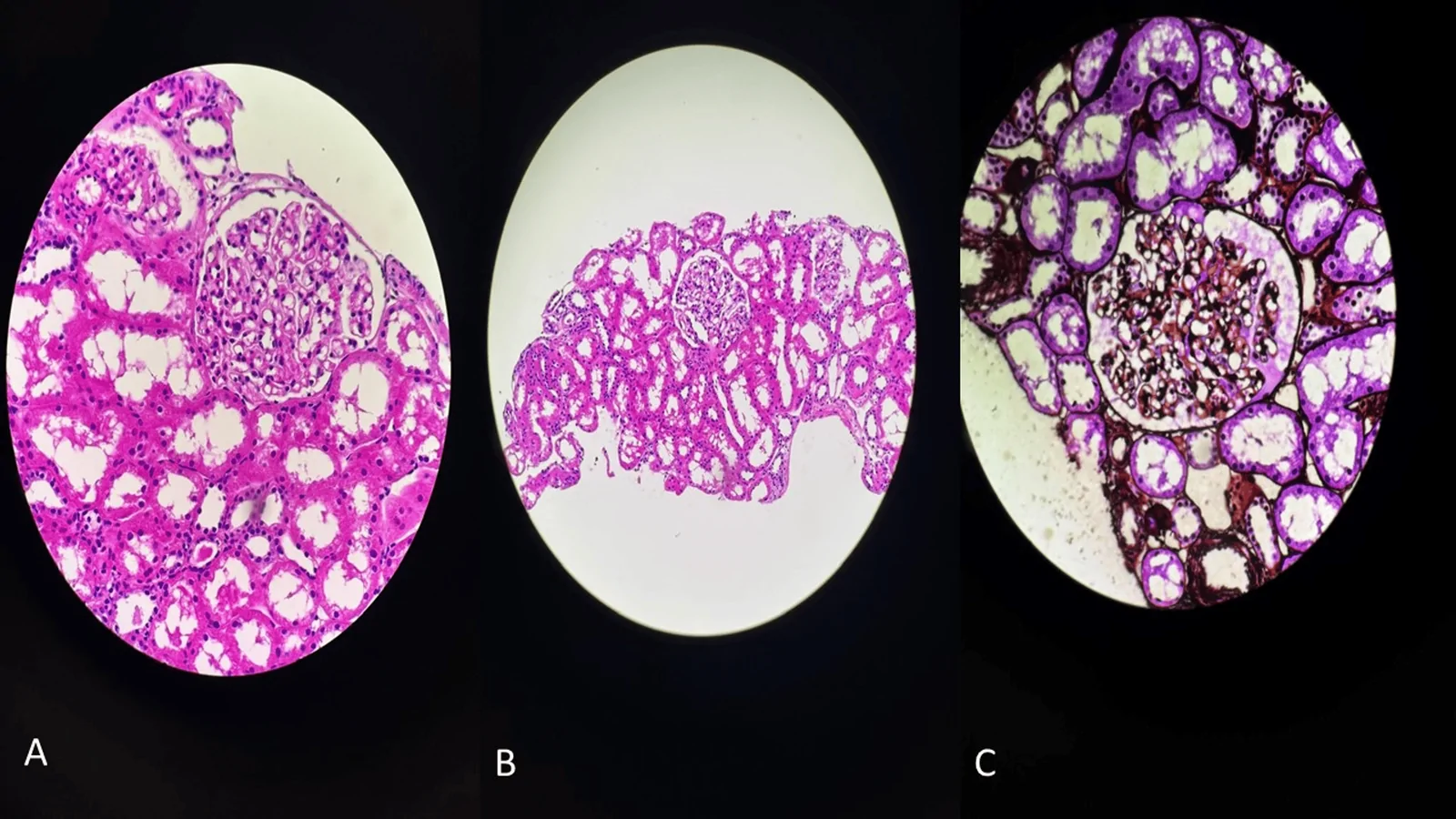
Representative light microscopy findings in Case 4 (A) High-power hematoxylin and eosin (H&E) staining (×400) showing a glomerulus with diffuse increased cellularity. (B) Low-power H&E staining (×100) showing a broader area of renal cortex with glomerular and tubular structures. (C) Reticulin staining (×200) highlighting the glomerular architecture and capillary loops.

The patient remained clinically stable following kidney biopsy and was discharged on ramipril therapy. She was subsequently referred back to the Department of Internal Medicine for further evaluation of her chronic inflammatory syndrome. Because follow-up was continued in another department, subsequent clinical and laboratory data, including serum creatinine, proteinuria, microscopic hematuria, serum complement levels, and additional immunological investigations, were unavailable. Consequently, although the clinicopathological findings favored an infection-related process, C3 glomerulopathy could not be formally excluded.

The main clinical characteristics of the four patients are summarized in Table 1.

**Table 1:** Clinical characteristics of the four patients AKI, acute kidney injury; ACE, angiotensin-converting enzyme; C3, complement component 3; C4, complement component 4.

Characteristic	Case 1	Case 2	Case 3	Case 4
Age (years)	36	31	43	46
Sex	Male	Female	Male	Female
Medical history	Type 1 diabetes mellitus	Recurrent pharyngitis	None	None
Suspected infectious trigger	Lower respiratory tract infection + Staphylococcus aureus urinary tract infection	Remote recurrent pharyngitis	Acute pansinusitis	Undetermined chronic inflammatory syndrome with erythematous lower-limb skin lesions
Clinical presentation	AKI with nephrotic syndrome	Persistent microscopic hematuria and proteinuria	Rapidly progressive AKI	Persistent microscopic hematuria and proteinuria
Blood pressure (mmHg)	130/69	149/91	120/65	124/65
Serum creatinine (µmol/L)	289	49	361	62
Urine protein-to-creatinine ratio (g/g)	2.99	0.8	2.2	1.2
Microscopic hematuria	Yes	Yes	Yes	Yes
Serum C3	Low (0.11 g/L)	Normal	Normal (1.27 g/L)	Not available
Serum C4	Low (0.06 g/L)	Normal	Normal (0.21 g/L)	Not available
Main treatment	Antibiotics + supportive care	Supportive care + ramipril	Supportive care; ACE inhibitor introduced after renal improvement	Supportive care + ramipril
Corticosteroids / immunosuppression	No	No	No	No
Outcome	Complete renal recovery	Persistent urinary abnormalities	Near-complete renal recovery	Transferred to the Department of Internal Medicine; long-term renal follow-up unavailable

## Discussion

Adult IRGN differs substantially from classic post-streptococcal glomerulonephritis described in children, with differences in epidemiology, clinical presentation, pathological features, and renal prognosis. IRGN may develop during an active infection rather than exclusively after its resolution [1,2]. Adult IRGN is increasingly recognized, particularly among individuals with diabetes mellitus, advanced age, chronic comorbidities, or immunocompromised states, with *Staphylococcus aureus *among the commonly implicated pathogens [1,2]. Clinical presentation ranges from isolated urinary abnormalities to severe AKI, nephrotic syndrome, and rapidly progressive glomerulonephritis [1,2].

The pathogenesis of IRGN involves complement activation in response to infection. Immunofluorescence typically demonstrates C3-dominant deposition, often accompanied by immunoglobulins [1,4,5]. However, predominant C3 deposition may be encountered in delayed or partially treated infection-related disease, creating substantial diagnostic overlap with C3 glomerulopathy [4-6]. In contrast, it is characterized by dysregulation of the alternative complement pathway, which may result from acquired autoantibodies or genetic abnormalities affecting complement regulation [5,6].

Our four cases illustrate the broad clinicopathological spectrum of adult glomerulonephritis with predominant C3 deposition in the setting of suspected infection-related disease. Case 1 provided the strongest evidence for active IRGN, with documented *Staphylococcus aureus* infection, marked hypocomplementemia, diffuse endocapillary proliferative glomerulonephritis, clinically defined nephrotic syndrome, and complete renal recovery during supportive management. Case 3 represented a more severe phenotype, with rapidly progressive AKI, crescentic glomerulonephritis, and predominant C3 deposition in the setting of recent acute pansinusitis. Despite the severity of the renal lesions, autoimmune investigations were negative; the biopsy favored a post-infectious origin, and kidney function progressively improved without corticosteroids, immunosuppressive therapy, or dialysis. In contrast, Cases 2 and 4 were characterized by preserved kidney function, persistent microscopic hematuria, and subnephrotic proteinuria. In both patients, kidney biopsy demonstrated predominant C3 deposition without a clearly documented active infection at the time of biopsy, leaving C3G as an important differential diagnosis. The main histopathological findings are summarized in Table 2.

**Table 2:** Histopathological findings of the four patients in the present case series C3G, C3 glomerulopathy; GN, glomerulonephritis; IRGN, infection-related glomerulonephritis.

Histopathological feature	Case 1	Case 2	Case 3	Case 4
Light microscopy	Diffuse endocapillary proliferative GN with extracapillary proliferation	Diffuse mesangial proliferative GN with exudative features	Crescentic GN with focal necrosis, diffuse mesangial sclerosis, and severe tubulointerstitial inflammation	Diffuse mesangial proliferative GN
Endocapillary proliferation	Yes	No	No	No
Mesangial changes	Not specifically assessed	Diffuse	Diffuse mesangial sclerosis	Diffuse
Crescents	Focal (2 glomeruli)	No	Circumferential (2 glomeruli)	No
Tubulointerstitial lesions	Mild inflammatory injury (<10%)	Mild fibrosis (~5%)	Severe inflammatory fibroedema	Mild fibrosis (<5%)
Vascular lesions	None	None	None	None
Immunofluorescence	C3-dominant mesangial deposition	C3-dominant mesangial deposition	C3 deposition in the mesangium and along the capillary walls	C3-dominant mesangial deposition
IgG	Trace	Negative	Negative	Negative
IgA	Negative	Negative	Negative	Negative
IgM	Negative	Trace	Negative	Trace
C1q	Negative	Negative	Negative	Negative
Pathological interpretation	Post-infectious GN	Remote/resolving IRGN; C3G not excluded	Probable post-infectious GN	Early or late IRGN; C3G not excluded

Adult IRGN encompasses several clinicopathological patterns and infectious settings. Endocarditis-associated glomerulonephritis represents an important infection-related form and should be considered when clinical features suggest an endovascular infectious source [9]. In our Case 1, transthoracic echocardiography showed no evidence of infective endocarditis. C3G is characterized by predominant C3 staining with absent or only limited immunoglobulin staining, and electron microscopy may help distinguish C3 glomerulonephritis from dense deposit disease [10]. Acquired and genetic abnormalities affecting the alternative complement pathway may contribute to the pathogenesis of C3 glomerulopathies [11]. Other variants of adult infection-related glomerulonephritis, including IgA-dominant post-infectious glomerulonephritis, have also been described [12]. Recent reviews emphasize the heterogeneous infectious, clinical, and pathological spectrum of IRGN in adults [13]. In older adults, diabetes, malignancy, staphylococcal infection, and pneumonia or urinary tract infection have been associated with adult post-infectious glomerulonephritis, although these findings primarily derive from cohorts of patients aged 65 years or older [14].

Our four cases similarly demonstrated diverse histopathological patterns, ranging from diffuse endocapillary proliferation to mesangial proliferative and crescentic lesions. Hypocomplementemia was documented in Case 1, whereas serum complement levels were normal in Cases 2 and 3, and subsequent complement assessment was unavailable in Case 4. Therefore, normal serum complement levels should not be considered sufficient to exclude IRGN and should be interpreted together with the infectious history, microbiological findings, kidney biopsy, and subsequent clinical course [1,2,14].

The principal diagnostic challenge in our series was distinguishing IRGN from C3G. Both disorders may present with hematuria, proteinuria, AKI, and predominant C3 deposition on immunofluorescence [4-6,10]. Histopathological findings alone may therefore be insufficient to establish the diagnosis. Predominant C3 deposition should be regarded as a histopathological pattern rather than a disease-specific finding [4-6,10]. Electron microscopy may provide additional information, particularly through identification of subepithelial hump-shaped deposits in infection-related disease and characterization of dense deposit disease within the C3G spectrum [4,5,8,10]. Unfortunately, electron microscopy was unavailable in our setting, representing an important limitation.

A documented or strongly suspected infection, an appropriate temporal relationship between infection and glomerular injury, improvement following treatment of the infectious process, and favorable renal evolution support an infection-related process. Conversely, persistent or recurrent urinary abnormalities, persistent complement abnormalities, progressive kidney dysfunction, or recurrence in the absence of an ongoing infectious trigger should raise suspicion for C3G and prompt additional complement evaluation when available [5-7,10,11]. Such investigations may include functional complement assays, complement autoantibody testing, genetic studies, and electron microscopy [5,6,10,11].

Cases 2 and 4 represented the greatest diagnostic uncertainty. In Case 2, the history of recurrent pharyngitis made a remote or resolving infection-related process clinically plausible, but persistent proteinuria and hematuria, together with the absence of definitive microbiological and complement investigations, prevented exclusion of C3G. Similarly, in Case 4, the inflammatory syndrome and kidney biopsy findings were compatible with an infection-related process, but the absence of longitudinal nephrological follow-up and advanced complement investigations prevented definitive exclusion of C3G. These cases emphasize the importance of avoiding an overconfident diagnosis based solely on C3 deposition and of integrating clinical context, disease course, and complementary investigations whenever available.

The contrasting outcomes of Cases 1 and 3 are also noteworthy. Both patients had substantial renal impairment but achieved marked renal recovery without corticosteroid or other immunosuppressive therapy. In Case 1, the documented bacterial infection, hypocomplementemia, characteristic endocapillary proliferation, and normalization of complement and urinary abnormalities strongly supported IRGN. In Case 3, the temporal relationship with recent acute pansinusitis, negative autoimmune investigations, biopsy interpretation favoring a post-infectious origin, and progressive renal recovery supported the same diagnosis despite crescentic glomerulonephritis. These observations illustrate that severe renal presentations, including crescentic lesions, may occur within the spectrum of infection-related disease [1,2].

From a practical perspective, predominant C3 deposition on kidney biopsy should not automatically lead to a diagnosis of C3G. The diagnostic approach should integrate the patient's infectious history, microbiological investigations, serum complement profile, kidney biopsy findings, and subsequent clinical course [4-7,10,11]. This distinction is particularly important because IRGN and C3G differ in pathophysiology, therapeutic approach, and prognosis. In our series, Cases 1 and 3 strongly favored IRGN, whereas Cases 2 and 4 remained diagnostically indeterminate because advanced complement investigations and electron microscopy were unavailable and, in Case 4, long-term nephrological follow-up was incomplete.

This study has several limitations. First, it was a retrospective single-center case series including only four patients. Second, electron microscopy was unavailable, precluding assessment of ultrastructural features that might assist in distinguishing IRGN from C3G. Third, comprehensive complement investigations, including functional complement assays, autoantibody testing, and genetic analyses, were not routinely available. Fourth, the original chest radiograph for Case 1, as well as the original renal CT images and renal biopsy slides for Case 3, were unavailable at the time of manuscript revision, although the corresponding radiological and histopathological reports were available. Finally, long-term follow-up was incomplete in one patient because subsequent care was continued in another department. These limitations should be considered when interpreting our findings.

Despite these limitations, the series provides detailed clinicopathological correlation across a heterogeneous spectrum of adult glomerulonephritis with predominant C3 deposition. All patients underwent kidney biopsy with light microscopy and immunofluorescence, while the clinical context and subsequent renal course provided additional information for diagnostic assessment. The favorable renal recovery observed in the patient with crescentic glomerulonephritis without immunosuppressive therapy also illustrates the importance of considering an infection-related etiology before initiating immunosuppression.

Overall, our findings support an individualized diagnostic approach in adults presenting with predominant C3 deposition on kidney biopsy. Careful integration of clinical history, microbiological evidence, complement studies, histopathological findings, and follow-up data is essential to distinguish IRGN from C3G. In settings where electron microscopy and advanced complement investigations are unavailable, clinicopathological correlation remains particularly important. Future multicenter studies incorporating electron microscopy, comprehensive complement profiling, and genetic analyses may help better define the diagnostic boundaries between these entities.

## Conclusions

Adult IRGN with predominant C3 deposition encompasses a broad clinicopathological spectrum and may closely mimic C3 glomerulopathy. Our four-case series illustrates that predominant C3 deposition is not specific for C3 glomerulopathy and should be interpreted in conjunction with the infectious history, microbiological investigations, serum complement profile, kidney biopsy findings, and clinical course. Severe renal presentations, including crescentic glomerulonephritis, may achieve favorable renal recovery with treatment of the underlying infection and supportive management. In cases where infection is remote, partially treated, or not definitively documented, C3 glomerulopathy should remain in the differential diagnosis. Clinicopathological correlation is therefore essential, particularly when electron microscopy and advanced complement studies are unavailable.
